# A large-scale model of the three first stages of the mammalian olfactory system implemented with spiking neurons

**DOI:** 10.1186/1471-2202-12-S1-P185

**Published:** 2011-07-18

**Authors:** Bernhard Kaplan, Simon Benjaminsson, Anders Lansner

**Affiliations:** 1 Department of Computational Biology, Royal Institute of Technology, Stockholm, S-10044, Sweden; 2 Department of Computational Biology, Stockholm University, Stockholm, S-11421, Sweden

## 

In this work we present a large-scale three stage model of the early mammalian olfactory system, including the olfactory epithelium (OE), the olfactory bulb (OB) and the olfactory (piriform) cortex (OC). All neurons in the network are modeled with a single or few compartments using the Hodgkin-Huxley formalism and are implemented in the NEURON simulator for parallel execution [1,2]. We investigate the dynamics of the network response to odorants and its performance in odor classification experiments.

The OE model comprises families of olfactory receptor neurons (ORNs) with different sensitivities, each family expressing one type of olfactory receptor (OR) with a vector of affinity values for each ligand [3]. These different ORN families connect to distinct glomeruli and mitral cells (MT) according to a hypothesized wiring scheme to form a fuzzy interval code for odorant concentration in the OB, i.e. each MT cell responds within a certain range of odorant concentration and these ranges overlap for different MT cells within one glomerulus. Mitral cells in different glomeruli respond independently to an odourant forming a sparse and distributed code in the OB, i.e. only a fraction of MT cells in different glomeruli is active when an odorant is present. The OC is modeled by a modular attractor network of pyramidal cells and inhibitory interneurons [4]. The OB response patterns are used to self-organize a projection to the OC based on learning algorithms employing ideas from machine learning (mutual information between the MT responses, multi-dimensional scaling, vector quantization and Hebbian learning) [5,6]. As a result, odourant responses are represented by a sparse distributed code in the OC.

**Figure 1:**
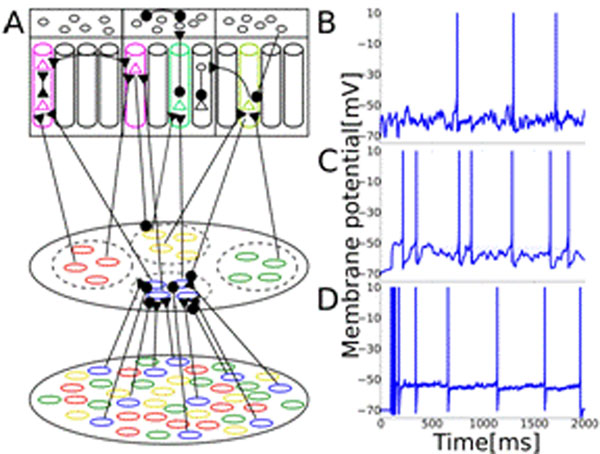
Schematic of the model (A) and sample membrane potentials of a pyramidal cell (B), a mitral cell (C) and an olfactory receptor neuron (D)

Results from runs with network sizes comprising thousands of model neurons show that this biophysically plausible network model generates response patterns of cells reminding of their real counterparts (see Figure 1), produces attractor dynamics in the olfactory cortex, and is able to discriminate between the different trained odors. We investigate effects of the model size, backprojections from OC to OB, study the performance of the model in discriminating mixtures of odorants and compare Calcium concentration in the olfactory cortex with experimental measurements [7].
